# Digital Twin Technology: The Future of Predicting Neurological Complications of Pediatric Cancers and Their Treatment

**DOI:** 10.3389/fonc.2021.781499

**Published:** 2022-01-19

**Authors:** Grace M. Thiong’o, James T. Rutka

**Affiliations:** ^1^ Division of Neurosurgery, Hospital for Sick Children, Toronto, ON, Canada; ^2^ Department of Surgery, University of Toronto, Toronto, ON, Canada

**Keywords:** digital twin (DT), prediction, neurologic sequelae, children and adolescents, cancer treatment, emerging technologies

## Abstract

Healthcare technologies have seen a surge in utilization during the COVID 19 pandemic. Remote patient care, virtual follow-up and other forms of futurism will likely see further adaptation both as a preparational strategy for future pandemics and due to the inevitable evolution of artificial intelligence. This manuscript theorizes the healthcare applications of digital twin technology. Digital twin is a triune concept that involves a physical model, a virtual counterpart, and the interplay between the two constructs. This interface between computer science and medicine is a new frontier with broad potential applications. We propose that digital twin technology can exhaustively and methodologically analyze the associations between a physical cancer patient and a corresponding digital counterpart with the goal of isolating predictors of neurological sequalae of disease. This proposition stems from the premise that data science can complement clinical acumen to scientifically inform the diagnostics, treatment planning and prognostication of cancer care. Specifically, digital twin could predict neurological complications through its utilization in precision medicine, modelling cancer care and treatment, predictive analytics and machine learning, and in consolidating various spectra of clinician opinions.

## Introduction

Digital twin is a concept that links a physical construct to its identical virtual counterpart *via* operations connecting the two entities. Ideally, the virtual domain replicates the behavior of the physical construct providing real-time data feedback and resulting in opportunities for timely resolution of errors. Ultimately, the dynamic entity should thrive sustainably through incorporating the digital intel and iteratively improving the physical model. The novelty of digital twins is evidenced by the less than 100 published manuscripts in the last decade, however, its versatile applications make it a frontier technology with widespread potential impact. Digital twin technology is the evolutionary outcome of Artificial Intelligence (AI), introduced in 1956 (1). NASA’s Apollo program pioneered the use of twin rovers by executing a similar concept of ‘mirror worlds’ described in 1991 (2–4). Co-opting the term ‘digital twin’ in healthcare would instinctively mean categorizing patients with a specific disease as the physical construct and data from diagnostics as a virtual domain. Today, digital twin technology has a myriad of applications in the manufacturing, healthcare and engineering industries (5). As the lexicon diversifies, from AI to ‘mirror worlds’ to ‘digital twin’, so do the applications. Currently, AI spans both the computer science and the physical engineering spaces. It is therefore not unimaginable to extrapolate digital twin solutions in surgical simulation, precision medicine and disease prediction modelling (6).

**Figure 1:**
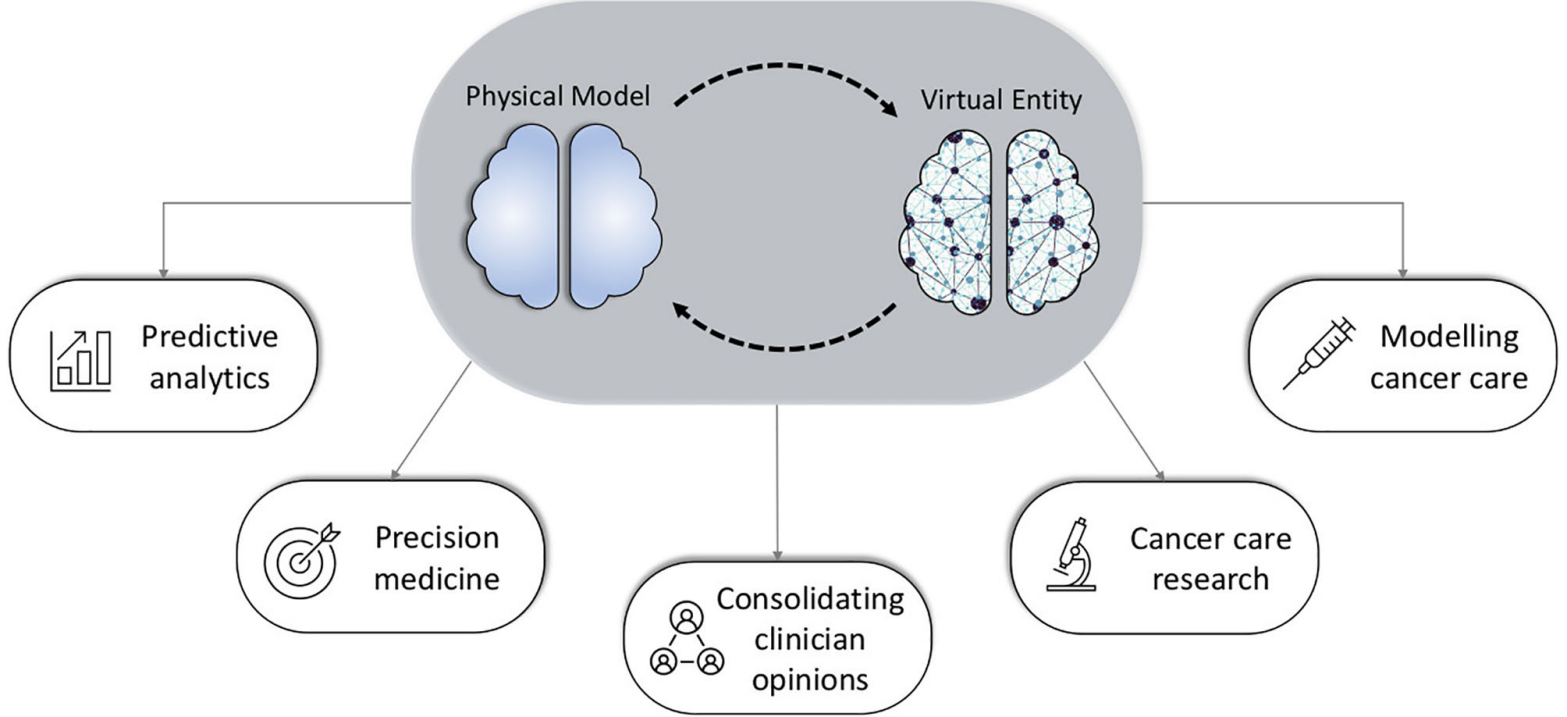
An illustration of the digital twin concept and it’s proposed applications in pediatric cancer care.

Actualizing digital twins for cancer treatment could be executed through seamless integration with already existing health infrastructure such as electronic health record (EHR) systems. Existing clinical data would be harnessed, and output maximized through additional plug-in software similar to how one would update applications or a new security feature on a computer. Further, innovative data collection strategies include continuous monitoring through health bracelets and watches, which simultaneously measure certain aspects of human physiology. The early adaptation of digital twin technology for specific uses in healthcare has stemmed from the incorporation of data from wearable sensors and tracking bracelets in a bid to augment spot-check clinical data points (7). Data have been compared to ‘currency’ in modern day research, consequently readily available data points are invaluable in developing predictive models and prognostic tools that would maximize desirable patient outcomes (8). More healthcare institutions globally will likely expand their adaptation of digital tools and technologies in order to minimize the risk of exposure to patients and clinicians without compromising patient care as exemplified by lessons from the COVID 19 pandemic (9). By citing specific examples of digital twin application in medicine, this manuscript discusses five clinically relevant themes for the pediatric cancer patient (summarized in **Figure 1**). Shortcomings associated with digital twin technology are mentioned in the concluding segment.

## Precision Medicine

Learning the specifics of an individual child’s neurologic complications currently entails the creation of single point data sets built on separate clinic visits, radiologic imaging, or other diagnostic occurrences over time. Digital twins developed around individual pediatric patients could personalize cancer care through modelling patient-specific risk predictors helpful in formulating prevention strategies or cures (10, 11). Wickramasinghe et al. illustrate from literature how three categories of digital twin technology: grey box, surrogate box and black box twins, can cohesively inform the diagnosis, data generation and treatment planning of uterine cancer patients (11). The principles, nevertheless, can be applied in other fields of such as in the context of pediatric cancer. Grey box twin models, that rely on existing knowledge of a patient, would likely be the easiest to incorporate as hospital EHR infrastructure is rife with clinical, laboratory and imaging data. Surrogate box twin models would, in addition, incorporate patient data from expanded clinical networks (11). Expanded networks encompass subspecialties in different wings or buildings of a hospital, different provinces, or even different countries involved in a consultative, diagnostic or treatment capacity of a single patient. The success of surrogate digital twin networks of a child with medulloblastoma, for example, whose surgery is performed in his/her home country and proton beam therapy delivered in a different country would depend on the digital connectivity and data exchange protocols between the two hospitals in two different countries. Black box digital twins, unlike grey and surrogate box models, do not rely on prior knowledge of physicians or patients. These cryptic models involve an element of deep learning (machine learning methods based on supervised, semi-supervised or unsupervised computational modelling) to generate patterns unique to a patient entity. The newly generated data, if applied in pediatric cancer care, would be predictive in nature and prognostic of neurologic complications.

## Modelling Cancer Care

Disease modelling through pooling cumulative data is one way that digital twins could predict the neurologic complications of pediatric cancers. Cumulative effects of novel treatments would be observed from small numbers of different patients globally. Justifiable geo-political or ethical constraints limit the number of pediatric patients enrolled into drug trials (12). Digital twinning could be the bridging platform for real-time analysis of the earliest pediatric cancer patient cohorts in leading research hospitals across the globe. Just as NASA gathers intel from its twin rovers, one on earth and the other on planet Mars, physicians and researchers could simultaneously analyze the physical patients recruited in trials and their corresponding digitized twin models for neurological sequelae. The resulting close and continuous monitoring would confer scientific rigor and yield early adverse effect reports. In this way digital twin modelling can augment limited clinical trial data and make it more robust (13). Robust virtual replicas of a clinical entity include a virtual liver description by Subramanian et al. (6) In this publication digital twin modelling investigated the complex interactions between liver functions, liver disease and the effect of drugs. Promising insights were gained that could be extrapolated to drug discovery and development research involving other pediatric cancer biologic systems.

### Modelling Cancer Research

Typically, research advances in drug discovery are achieved through well conducted clinical trials often with strict eligibility protocols. This stringency precludes from analysis data obtained through the compassionate use of the same drugs. Compassionate use of novel treatments, for reasons such as the neurological sequalae of pediatric cancers, coupled with digital twinning could arguably increase data points for research analysis and reporting (13). In fact, when applied on a large scale basis, Dov Greenbaum proposes that digital twins could supplement randomized control trials (13). This supplementation could happen by tapping into ‘Big Data’ (data from multiple sources) through twinning models which in a few studies have shown promise, over traditional man-made models, in identifying novel predictors of disease (14, 15).

Perhaps, in a distant future, levels of scientific evidence would have to be redefined to reflect the robustness that digital twin data would inject into well designed randomized control trials. In the meantime, a more direct application of digital twins for the pediatric cancer population would be their utilization in bench side research. Individual mouse models for a myriad of pediatric disease currently exist for the advancement of research, understanding the natural history as well as testing out new treatments. Digital twin by extrapolation could amplify observations made from mouse models. For example, a digital twin of the traditional medulloblastoma mouse model could uncover new predictors resulting from real-time observations of the physical mouse’s physiology in response to a certain intervention. Specific rare molecular subtypes of cancers could be studied iteratively between *in vivo* cancer models and their concomitant predictive algorithms resulting in deeper and faster comprehension of the natural history of disease.

## Predictive Analytics and Machine Learning

Pediatric cancers are a diverse field encompassing three spheres: diagnostics, a repertoire of treatment options and lengthy patient follow-up. The multiple data points thus generated across time and space make this field the ideal case study for the convergence of digital twin and machine learning technologies. The intersection between statistics and computer science is known as predictive analytics. Frequently, machine learning algorithms are applied to the analysis of the complex interactions within big data in order to extract useful patterns therein (16). In healthcare research this technology could be used to extract clinically relevant predictive patterns of disease. Vast amounts of data currently exist and continue to accumulate owing to a global commitment to cancer research. Predictive analytics and machine learning are well suited to encompass the multiple point data embedded within clinical practice and research (15). Literature has described machine learning’s greater success in cardiovascular risk prediction over expert clinicians prognostication (14). Similar success could likely be replicated in risk prediction and stratification of pediatric cancer neurologic complications. Owing to the readily available cumulative health data coupling digital twin technology with data mining through machine learning algorithms could well be the much needed avenue to discover specific predictors of disease (17).

## Consolidating Diverse Clinician Opinions

Judicious patient assessment and clinical judgement cannot be replaced by artificial intelligence. Clinicians form, perhaps, the most integral part of pediatric cancer care treatment. Various specialists are trained to practice standard of care and make recommendations based on astute clinical judgement. Standard of care often entails clear-cut decisions nevertheless; it is not unusual for differences to sometimes arise and for clinical opinions to differ among doctors. This spectrum of clinician treatment recommendations is exemplified by a study examining the differences between Japanese and Western pathologists in their diagnostic criteria of gastric cancers (18). The study concluded that not only did pathologist grading interpretation prevail among the cohort, but that the differences between pathologists was also of prognostic significance. Self-learning digital twin models, over time, can assess the limits of assessment among different clinicians’ differences and hence contribute reducing the margin of error. Rao et al. has used digital twin technology to demonstrate how these differences in opinion can be mitigated in what the authors describe as ‘collating the subjective risk’ (19). In this example, the applied digital twin model isolated new patterns based on the interactions between specified knowledge constraints and diverse datasets including physician input. Patterns learned through data science can thus consolidate diverse clinician opinions and offer a consensus if needed.

## Conclusion

In spite of the unequivocal contribution that artificial intelligence has,so far, made in healthcare and the potential applications that digital twin can have for the advancement of disease prediction it is prudent to mention the associated shortcomings (14). To begin with, the ethics of artificial intelligence revolving around privacy and access to the continuously generated patient health information should be carefully considered. Although digital twin is a relatively new concept, privacy and internet security are ongoing conversations across all fields. Nevertheless, the widespread installation of EHR systems in hospitals has already set precedence by placing access protocols for primary care physicians and verified patient circles of care. Secondly, the incorporation of electronic health recording or its expanded use would be an added cost to the overall healthcare of a cancer patient, as would the cost of new wearable technologies and the networking needed to complete the digital twin triad. Finally, some digital twin models, such as black box algorithms, are heavily reliant on deep learning computations and have been met with some criticism over the specifics of their inner workings (20). Perhaps, encouraging more clinicians to be involved in the formation of the background algorithms, in a consultative capacity with data engineers, could give more credence to the process.

In summary, in this manuscript proposes an emerging concept, digital twin technology, as a frontier strategy for the identification of predictors of neurological complications of pediatric cancers and their treatment. By exploring the interface between the physical cancer patient and artificial intelligence, healthcare researchers can begin to broach an inevitable future where the lines between man and machine will no doubt get blurry.

## Data Availability Statement

The original contributions presented in the study are included in the article/Supplementary Material. Further inquiries can be directed to the corresponding author.

## Author Contributions

Author GMT contributed as the first author, whereas author JTR contributed as the senior author of the manuscript. All authors contributed to the article and approved the submitted version.

## Conflict of Interest

The authors declare that the research was conducted in the absence of any commercial or financial relationships that could be construed as a potential conflict of interest.

## Publisher’s Note

All claims expressed in this article are solely those of the authors and do not necessarily represent those of their affiliated organizations, or those of the publisher, the editors and the reviewers. Any product that may be evaluated in this article, or claim that may be made by its manufacturer, is not guaranteed or endorsed by the publisher.
